# Atypical presentation of forearm compartment syndrome in a case of vascular type Ehlers–Danlos syndrome

**DOI:** 10.1080/23320885.2022.2158833

**Published:** 2023-01-20

**Authors:** Tsang Yeung, Ching San Esther Chow

**Affiliations:** Department of Orthopaedics and Traumatology, United Christian Hospital, Hong Kong

**Keywords:** Compartment syndrome, Ehlers–Danlos syndrome, Hong Kong Chinese, pseudoaneurysm, saphenous vein graft

## Abstract

A 30-year-old Chinese man with vascular type Ehlers–Danlos Syndrome presents with spontaneous right forearm compartment syndrome due to pseudoaneurysms along the radial artery. Emergency fasciotomy and reconstruction of the radial artery with a saphenous vein graft were performed. Genetic test showed a heterozygous DNA change c. 1852 G > C in COL3A1 gene.

## Case report

A 30-year-old Chinese man labelled as Ehlers–Danlos Syndrome (‘EDS’) due to positive family history, with the past history of epilepsy, admitted to our unit in the year 2017 with bilateral forearm redness and swelling for two days, with the right side being more severe. There was no recent history of epileptic convulsion, insect bite or trauma. The patient did not complain of any paraesthesia of both upper limbs.

On admission, he had normal blood pressure and was afebrile. His bilateral forearms were mildly erythematous with bruising, mildly tender and with an increase in temperature. His bilateral wrists movement was normal. White blood cell count was 17.2 × 10^9^/L and C-reactive Protein (CRP) was 190.1 mg/L. He was therefore treated as cellulitis initially with antibiotics.

**Figure 1.**
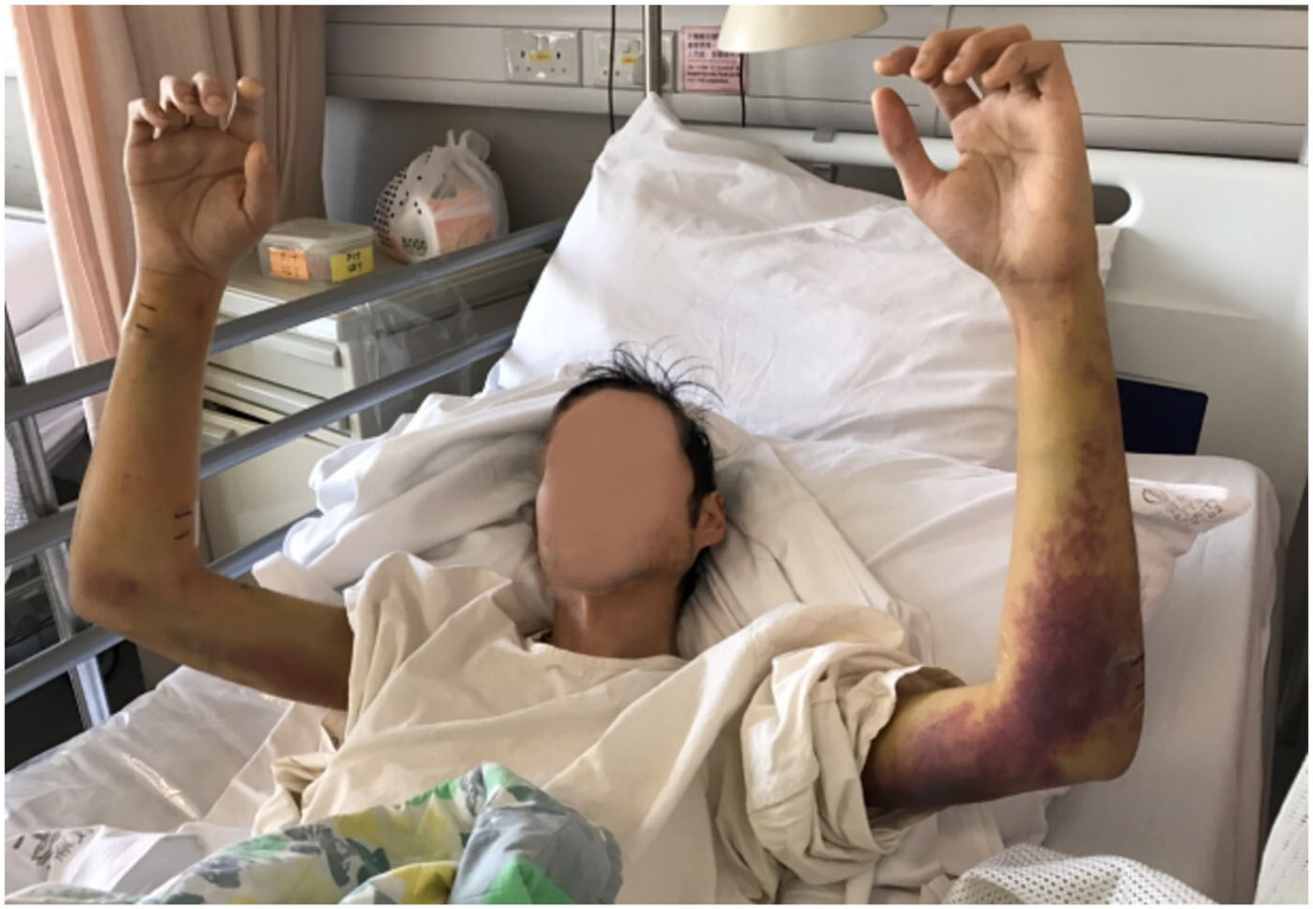
Clinical Photo of patient on admission. There was marked swelling, bruising over bilateral forearms.

During patient’s stay, progressive right forearm swelling was noted. Repeated physical examination found tense forearm compartments, and there was also limited right wrist and fingers movement; the radial pulse was weakened. Left forearm compartments were not tense. There was no numbness over bilateral upper limbs (Figure 1).

**Figure 2.**
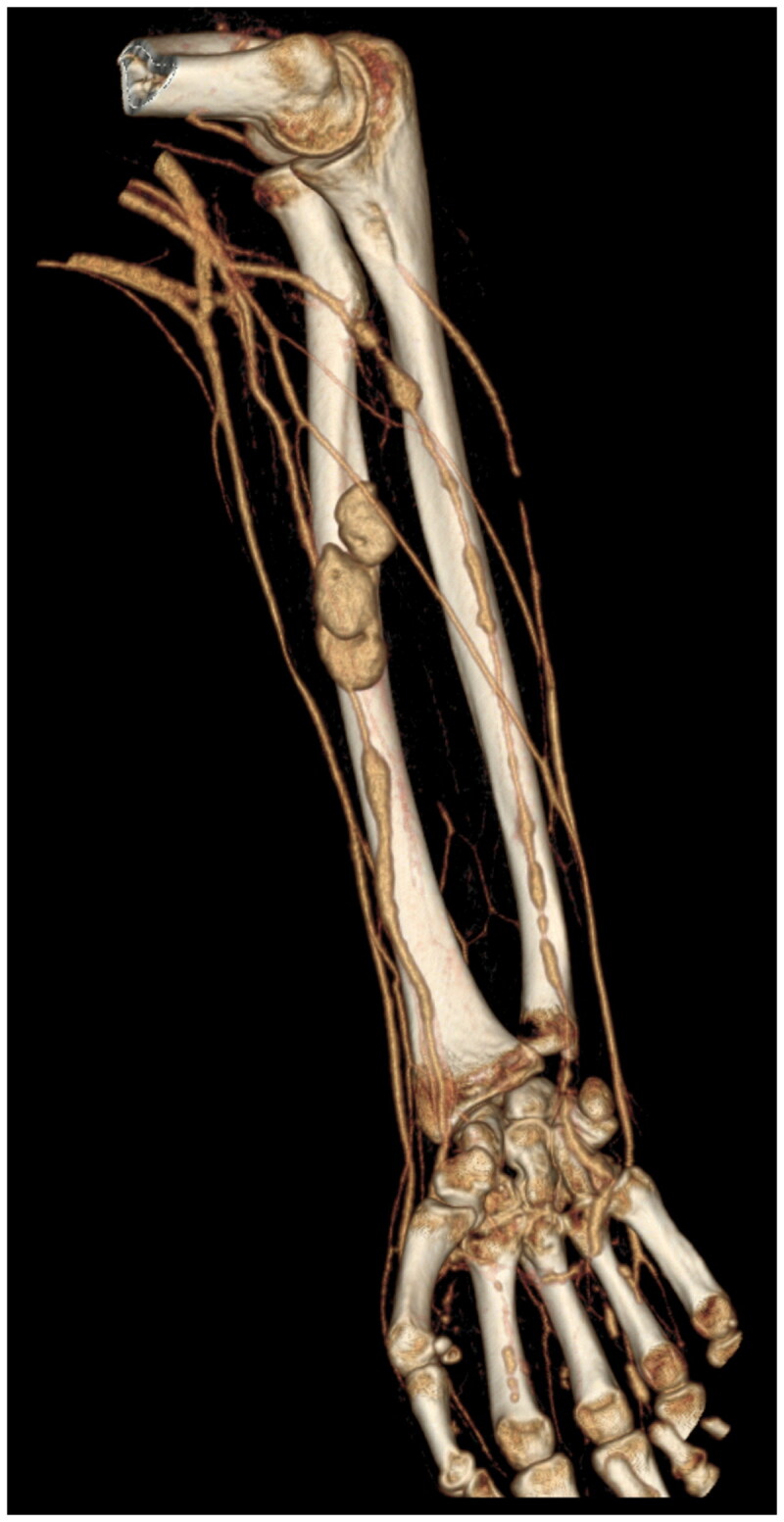
CT Angiogram showed multiple pseudoaneurysms along radial and ulnar arteries.

Compartment pressure was measured and found to be up to 62 mmHg. An urgent CT angiogram of the right forearm showed multiple pseudoaneurysms along radial and ulnar arteries (Figure 2).

**Figure 3.**
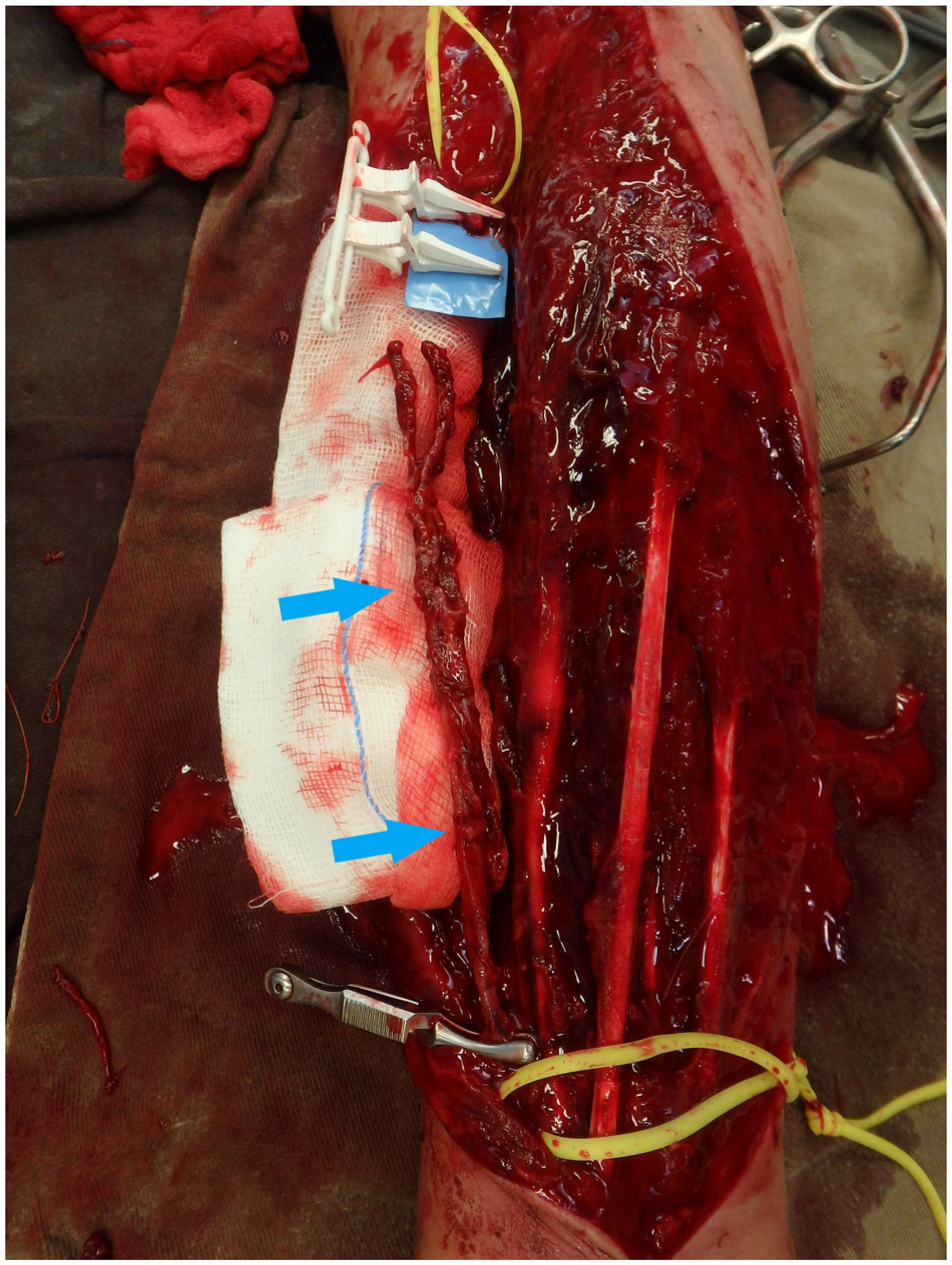
15 cm segment of the radial artery was found to be thinned-wall and friable, with multiple sites of rupture (Blue arrows).

**Figure 4.**
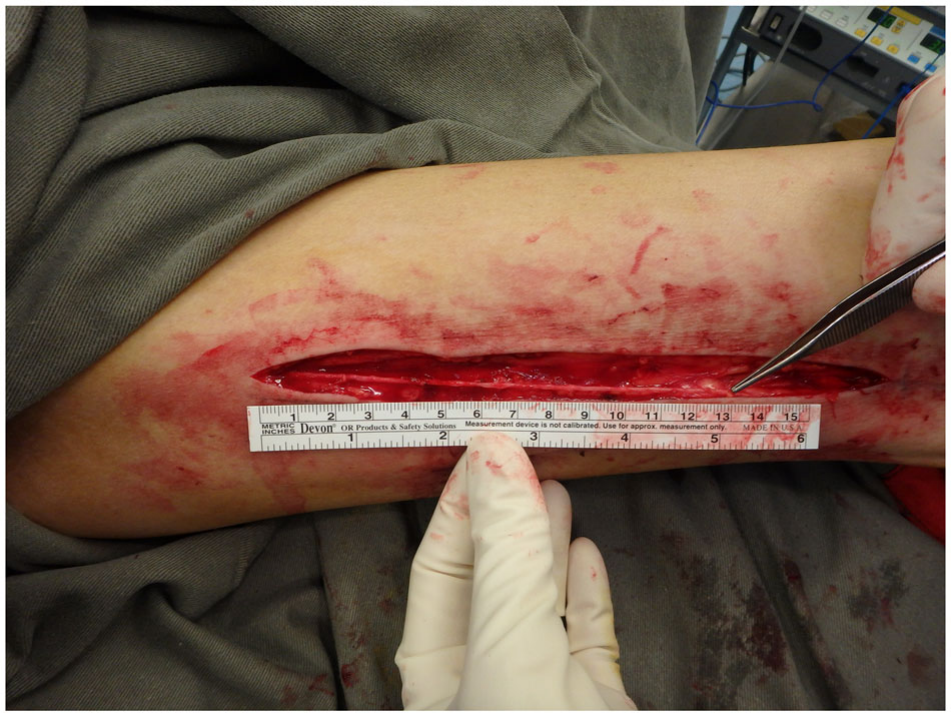
Saphenous vein graft was harvest from left medial thigh.

**Figure 5.**
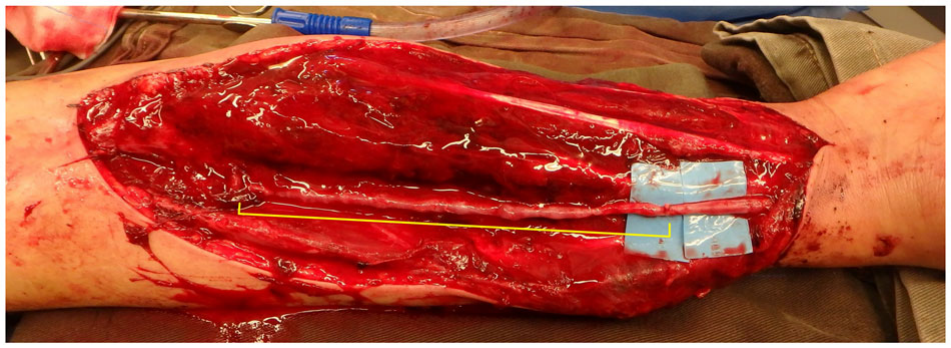
The vein graft was used to reconstruct the vascular defect (Yellow segment).

**Figure 6.**
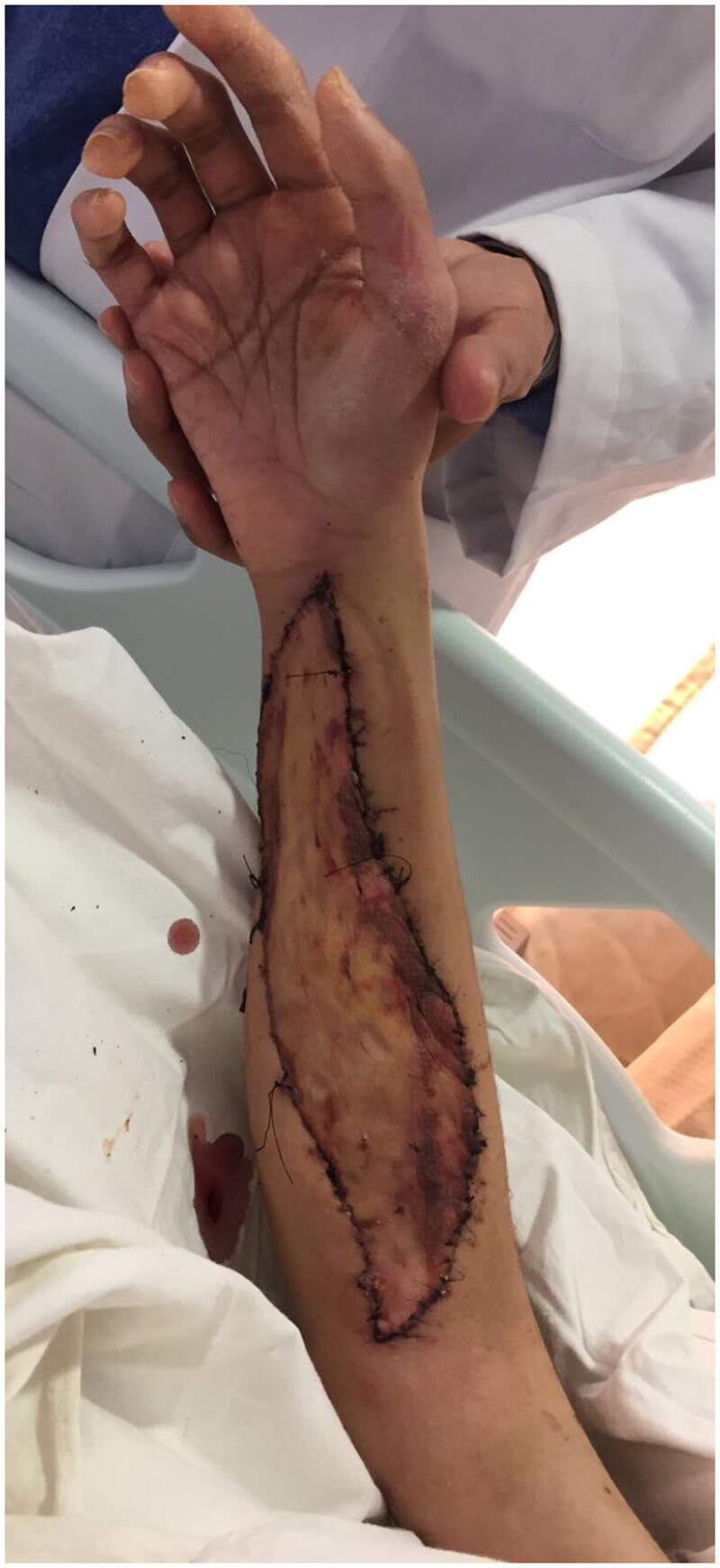
Clinical Photo of patient’s right forearm after skin grafting.

Urgent forearm fasciotomy was performed. Intraoperatively, a 15 cm segment of the radial artery was found to be thinned-wall and friable, with multiple sites of rupture (Figure 3). The pathological segment was excised and reconstructed with a saphenous vein graft (Figures 4 and 5). Circulation of the right upper limb was restored. The fasciotomy wounds were covered with full thickness skin graft six days after the index operation (Figure 6).

Microscopic examination by a pathologist found that the radial artery was with variable smooth muscle thickness, partial loss of muscle wall, aneurysmal dilatation, fibrinoid necrosis and pseudoaneurysm surrounded by a moderately dense mixed inflammatory infiltrate and fibroblastic reaction. There was an absence of internal elastic lamina and loss of muscle wall at the aneurysmal sites. There was also a variable decrease in elastic fibers and smooth muscle cells in the tunica media. Similarly, the adjacent small to medium-sized veins had focal deficient or fragmented elastic fibers noted in the tunica media.

Genomic investigation has been carried out and it confirmed that the patient has a heterozygous variant in the COL3A1 gene which is a DNA change c. 1852 G > C in exon 26.

**Figure 7.**
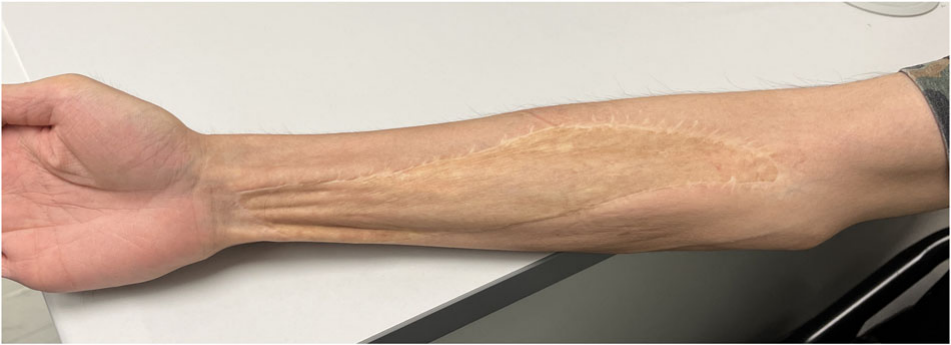
Four years after operation – the skin graft incorporated and the wound healed well.

It has been four years after the operation. Upon follow-up, the wound healed well and the patient has good right-hand function and he can write nicely with his right hand. To our knowledge, there were no complications and the reconstructed radial artery did not rupture (Figure. 7). The patient has consented to the publication of this manuscript.

## Discussion

Ehlers–Danlos syndrome (‘EDS’) is a group of hereditary connective tissue disorders, caused by various defects in the synthesis of collagen. Its overall prevalence lies between 1 in 10,000 to 25,000 in the general population [1]. EDS has different subtypes. Type IV or the vascular type Ehlers–Danlos Syndrome (‘vEDS’), described by Andras Barbaras [2] in 1967, consists of 5 to 10% of EDS [1]. It is due to pathogenic variants in COL3A1 gene and it is inherited in an autosomal dominant manner. Due to defects in type III collagen, body tissues and organs of patients with vEDS are fragile. Signs of vEDS include: characteristic facial appearance (thin vermilion of lips, small chin, thin nose, large eyes), acrogeria (skin on the hands and feet appears prematurely aged) and thin translucent skin [3]. In a majority of patients, the diagnosis was only made when there is at least one major complication happened, which is defined as arterial rupture, dissection or organ rupture, like sigmoid colon perforation and perforated gravid uterus The risk of having complication at age of 20 is 25% and rises to more than 80% by age of 40. The average age at the time of the first major complication was 23.5 years old. Median life expectancy of patient with vEDS is 48 years [4].

Prior to this case report, compartment syndrome as a complication of vEDS has also been reported in the literature from different countries. The first case was reported in 1992 with spontaneous bleeding from a gluteal artery resulting in a gluteal compartment syndrome and sciatic neuropathy [5]. There were also cases of abdominal compartment syndrome [6], compartment syndromes over the legs, upper arm and forearm due to rupture of peroneal artery [7], posterior tibial artery [8] and ulnar artery [9]. The current case is the first reported case of radial artery pseudoaneurysm.

The challenge in these cases of compartment syndrome in vEDS patient is that surgeons also need to tackle the pseudoaneurysms and maintaining adequate blood flow to the limbs apart from performing facsiotomy. Moreover, care has to be taken to avoid profuse bleeding from the fragile or ruptured major vessels. Surgical options of treating pseudoaneurysm were described in those case reports, such as endovascular coil embolization, oversewing of the ends of ruptured pseudoaneurysm with pledgeted suture, stenting, or ligation of the artery if another patent artery suppling the involved limb is present. Yet, endovascular intervention is not free of risk. Rupture and pseudoaneurysm formation at the access site [10] and arterial complication secondary to placement of the embolization coils [11] have been reported. Gentle, meticulous technique and careful soft tissue handling are utmost important to minimize intra-operative complications.

In order to reconstruct a long segment of a small caliber artery, vein autograft might be the only option, as a synthetic conduit of small caliber (d < 6mm) is rare in the market [12]. However, since the defect in collagen synthesis in vEDS would also cause abnormalities in veins, as evidenced by the histological finding of the veins specimens in our case, the long-term result is uncertain due to limited cases reported in the literature. While there was no complication seen after four years since the operation in our case, there was another case of saphenous vein graft disruption leading to haematoma formation and reoperation reported by Shalhub [13].

We aware that the major limitation of our case report is that a single case lacks the generalisability to confirm the safety and durability of auto-vein-grafting in patients with vEDS. We would therefore recommend further study of the higher level of evidence on the use of autologous vein graft with a longer duration of follow-up.

Last but not least, the DNA change c. 1852 G > C in the COL3A1 gene detected in this patient changed the 618th codon from Glycine to Arginine. It is classified as likely pathogenic by American College of Medical Genetics (ACMG) guidelines. This missense change has not been reported in the literature or Human Gene Mutation Database. A different change c. 1853 G > T in the same codon has been reported as disease-causing for vEDS phenotypes in the previous study [14].

## Conclusion

Although rare, acute limb compartment syndrome could happen spontaneously in a patient with vEDS. High index of suspicion of compartment syndrome, timely urgent fasciotomy and vascular intervention with meticulous technique and gentle soft tissue handling during operation are the keys to prevent complications. We believe that autologous saphenous vein grafting is a safe, durable option to reconstruct a long segment of an arterial defect in this group of patients. However, in view of underlying fragility of the patient’s own veins, further study is recommended to confirm the long-term outcome of using autologous vein graft in treating patients with vEDS.
